# Cohort Profile Update: Africa Centre Demographic Information System (ACDIS) and population-based HIV survey

**DOI:** 10.1093/ije/dyaa264

**Published:** 2021-01-12

**Authors:** Dickman Gareta, Kathy Baisley, Thobeka Mngomezulu, Theresa Smit, Thandeka Khoza, Siyabonga Nxumalo, Jaco Dreyer, Sweetness Dube, Nomathamsanqa Majozi, Gregory Ording-Jesperson, Eugene Ehlers, Guy Harling, Maryam Shahmanesh, Mark Siedner, Willem Hanekom, Kobus Herbst

**Affiliations:** 1 Africa Health Research Institute, KwaZulu-Natal, South Africa; 2 London School of Hygiene and Tropical Medicine, Faculty of Epidemiology and Population Health, London, UK; 3 Institute for Global Health, University College London, London, UK; 4 MRC/Wits Rural Public Health & Health Transitions Research Unit (Agincourt), University of the Witwatersrand, Johannesburg, South Africa; 5 Department of Epidemiology & Harvard Centre for Population and Development Studies, Harvard T.H. Chan School of Public Health, Boston, MA, USA; 6 Harvard Medical School, Boston, MA, USA; 7 Division of Infectious Diseases, Massachusetts General Hospital, Boston, MA, USA; 8 SAPRIN, South African Medical Research Council, Cape Town, South Africa

**Keywords:** South Africa, epidemiology, HIV, population cohort

**Key Features**

- The Africa Centre Demographic Information System (ACDIS) cohort in rural KwaZulu Natal, South Africa, was established in 2000 by the Africa Health Research Institute (AHRI). In 2017, the cohort was expanded and renamed the Population Intervention Platform (PIP).
- Public health priorities in South Africa have changed over the past 20 years, with widespread availability of antiretroviral therapy (ART) and a growing burden of non-communicable diseases (NCDs). AHRI’s research programme has shifted to address these new priorities, and to deliver and evaluate interventions.
- In mid-2018, the cohort had ∼140 000 individuals (median age = 23 years, interquartile range = 11–36), of whom 66% were members of households in the original ACDIS cohort.
- New questions include additional household socio-economic indicators, and diagnosis and treatment of HIV, tuberculosis (TB) and NCDs. Attendance at clinics in the PIP area is captured. New linkages to routine data provide information on treatment, morbidity, health service usage and a range of health outcomes.
- Data can be accessed through the AHRI data repository (https://data.ahri.org/index.php/home).

## The original cohort

The Africa Centre Demographic Information System (ACDIS) was established in 2000 by the Africa Health Research Institute (AHRI; formerly the Africa Centre for Health and Population Studies), funded by the Wellcome Trust. The aim of ACDIS was to describe the demographic, social and health impacts of a rapidly progressing HIV epidemic in rural South Africa, and to monitor the impact of intervention strategies. An initial cohort profile was published in 2008 and described findings from the first 6 years of data collection.^1^

**Figure 1:**
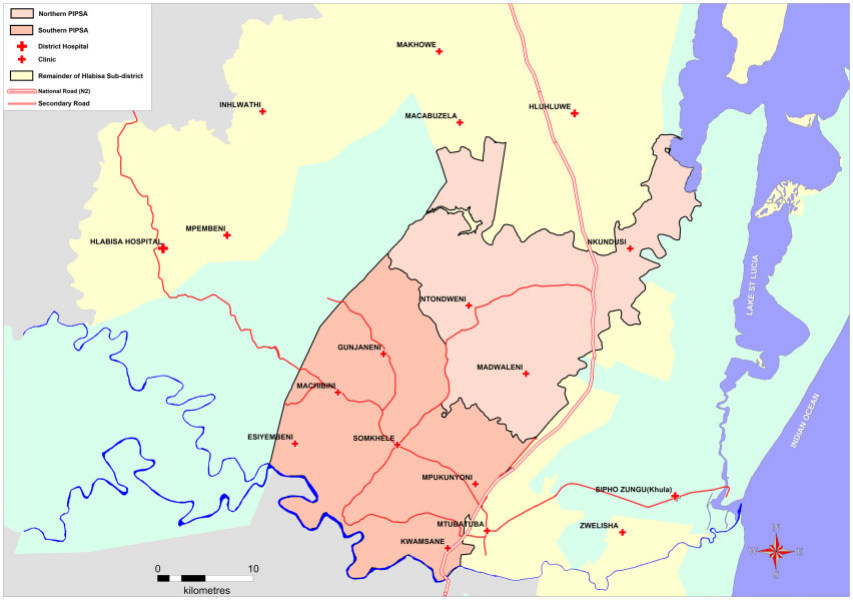
Map of the expanded surveillance area. The PIP includes the households of the original ACDIS surveillance area [southern PIP surveillancd area (SA)], and those of the recently completed Treatment as Prevention (TasP) trial (northern PIPSA)

The original ACDIS surveillance area covered 438 km^2^ in uMkhanyakude district, KwaZulu-Natal province, South Africa, and included a population of ∼85 000 resident and non-resident individuals in 11 000 households in 2006. Households are contacted three times annually to record information on births, deaths and migration patterns of all household members, including non-residents. Resident members aged ≥15 years are invited to participate in an annual individual-level survey, which includes an interview on general health and sexual behaviour, and collection of a dried blood spot (DBS) for anonymized HIV testing. Geographic coordinates are available for each homestead, allowing spatial analysis. In 2017, the ACDIS area was expanded to 845 km^2^ and renamed the Population Intervention Programme (PIP), with ∼140 000 individuals (20 000 households) in 2018, including the communities of a recent cluster-randomized trial (CRT) of HIV treatment (Figure 1).^2^

## What is the reason for the new focus?

ACDIS has made important contributions to our understanding of the HIV epidemic. However, despite significant increases in antiretroviral therapy (ART) coverage, HIV incidence remains high. South Africa has the largest number of people living with HIV worldwide, estimated at 7.7 million in 2018. In the PIP area, HIV incidence among women aged 15‒24 years in 2011‒2015 was estimated at 6.2/100 person-years.^3^ Incidence has declined more recently, with marked reductions between 2014 and 2017 among young women.^4^

Moreover, the HIV prevention landscape has changed considerably since the initiation of the original ACDIS cohort. The scale-up of ART (first rolled out in the surveillance area in 2004) has had a major impact on the health and life expectancy of HIV-positive individuals.^5^ However, new infections need to be prevented to bring an end to the HIV pandemic. Effective HIV prevention will require a combination of biomedical, behavioural and structural interventions that will need to be tested at the population level.

Aside from HIV, there is a rapidly changing burden of disease in the region. Tuberculosis (TB) is responsible for >30% of deaths in KwaZulu Natal, and the uMkhanyakude district has a high prevalence of rifampicin resistance (9.9% of TB cases vs 6.9% nationally in 2017).^6^ In parallel, South Africa is experiencing a growing burden of non-communicable diseases (NCDs), especially diabetes, cardiovascular disease, hypertension, kidney disease and cancer. Injuries (both traffic accidents and violence) are also an important cause of death.^7^ As the public health priorities in rural South Africa have shifted significantly over the past 20 years, our research programme has made a parallel shift to address these new priorities. In addition, our focus has changed from descriptive epidemiology to delivering and evaluating interventions.

Lastly, advances in surveillance methods have resulted in changes to our survey methodology. The collection of clinical data requires an efficient and reliable way of communicating results to individuals, and of linking those who require care to appropriate services. Advances in technology have also allowed us to introduce telephonic and electronic data capture.

## What will be the new areas of research?

AHRI continues to focus its research on HIV, and has extended to TB, NCDs and the interaction of chronic infections with NCDs. New areas of research will include the pathogenesis and prevention of HIV and TB, and the biological, structural and social reasons for continued HIV transmission despite widespread ART use. In October 2016, South Africa implemented the new WHO universal test and treat (UTT) guidelines, whereby ART is offered to all individuals living with HIV. With increasing numbers of individuals on ART, PIP allows exploration of host and viral determinants of responses to therapy and HIV disease progression, development of drug resistance mutations, the impact of drug resistance on ‘treatment as prevention’ goals, and interactions and comorbidities of HIV, TB and NCDs.

New research will also focus on the biological factors associated with susceptibility to HIV, including host microbiome and host and viral genetics, to inform new prevention approaches. Stored blood samples from the period before and after HIV acquisition in >3500 individuals provides an opportunity for genetic sequencing to explore biological markers of HIV susceptibility.

A new focus will be on the transmission dynamics of TB, including the origin of drug-resistant strains and identification of biomarkers that predict treatment success. PIP will also provide a platform for ancillary studies nested within the surveillance cohort. There will be an increased focus on interventions to reduce HIV and TB transmission, as well as interventions to improve access to essential health services.

## Who is in the cohort?

PIP is an open cohort consisting of all households within the expanded surveillance area. As with ACDIS, information is collected on both resident and non-resident members who join or leave the cohort at any given time (a resident is defined as an individual who intends to sleep the majority of nights in the homestead occupied by the household). In addition to the existing ACDIS cohort, PIP includes the communities of the ANRS 12249 Treatment as Prevention trial;^2^ households were followed in the trial from 2012 to 2016 and were added to the PIP cohort in 2017.

**Figure 2:**
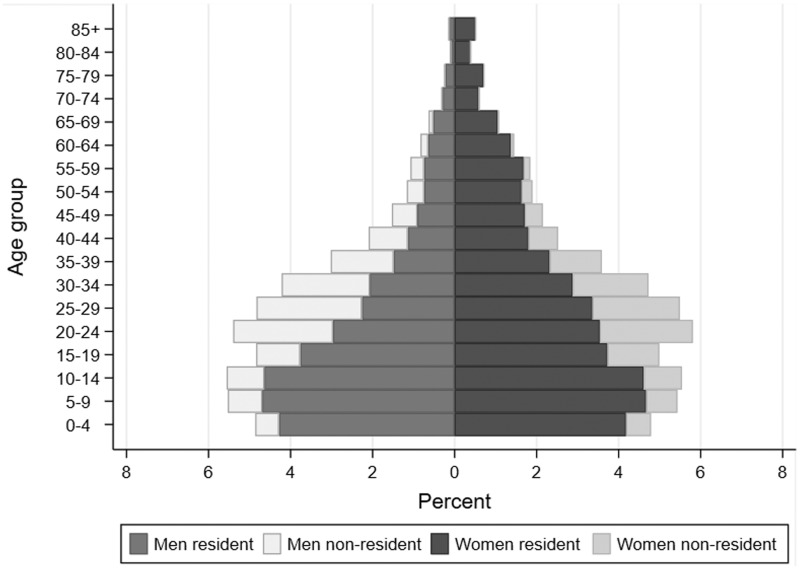
Age and sex profile of the surveillance population by residency status, 1 July 2018 (102 731 residents; 39 348 non-residents)

Since 2000, 231 179 unique individuals have participated in the cohort, generating >2 million person-years of follow-up. Only 5.2% of individuals have been lost to follow-up, although members may exit if they leave a member household. In mid-2018, there were 142 079 individuals in the PIP cohort, of whom 93 074 were members of households in the original ACDIS surveillance area (Figure 2; Supplementary Table S1, available as Supplementary data at *IJE* online). Overall, 54% (76 134) of the cohort were female and 28% were non-resident members.

Compared with the 2006 cohort ,^1^ the population is older: median (interquartile range) male and female ages were 22 years (11–34) and 25 years (12–39) respectively in mid-2018, vs 19 years (10–31) and 21 years (11–35) in mid-2006. Non-resident members make up a slightly lower proportion of the population (28% in 2018 vs 34% in 2006). Household size is smaller (mean = 7.0, SD = 4.4 in 2018 vs mean = 7.9, SD = 4.7 in 2006), and many socio-economic indicators have improved. Access to electricity and toilet facilities have increased to >95% of households; however, access to piped water has declined from 78% in 2006 to 66% in 2018. Unemployment remains high, with 62% of adults without formal employment (similar to 2006); however, receipt of government grants has expanded to 39% of household members. HIV prevalence has increased substantially, largely owing to access to ART and improved survival. Prevalence among resident men and women aged 15–54 years who provided an anonymous DBS in 2018 was 19 and 40%, respectively, vs 13 and 25% in 2006 (Supplementary Figure S1, available as Supplementary data at *IJE* online).

Participation in the household-level survey is extremely high (>98%), and has remained stable over time. Participation in the individual-level component in any given year is <50% (Supplementary Table S2, available as Supplementary data at *IJE* online). However, between 2003 and 2012, 68% of eligible individuals participated in the HIV surveillance at least once and 48% at least twice, after five survey rounds.^8^ Participation has increased more recently; between 2013 and 2018 these figures were 76 and 53%, respectively.

## What has been measured?

PIP continues to collect much of the information described in the original cohort profile. The main changes involve the following.

- Reduction in the number of home-based survey visits. Since 2017, visits to PIP households have been reduced to once annually, with telephone interviews twice annually to update demographic information.
- Capturing questionnaire data electronically and addition of new questions. Questionnaires are administered on tablet computers using the Research Electronic Data Capture (REDCap) system.^9^ Sexual behaviour and other sensitive questions are collected by computer-assisted self-interview. Questions have been added to the household survey on receipt of government grants, food security and experience of violence (Table 1). The verbal autopsy (VA) questionnaire (administered routinely for all deaths) has been updated to conform to WHO/InterVA standards. In the individual survey, questions have been added about diagnoses and treatment of HIV, TB, hypertension and diabetes. Most recently, COVID-19 surveillance has been introduced.^10^
- Offering home-based HIV counselling and testing (HCT) during the survey visit. At the annual home visit, all resident household members aged ≥15 years who are not on ART are offered HCT, even if they do not participate in the survey. Individuals who test positive are referred to HIV care at a clinic in the surveillance area. They are also asked to consent to facilitated linkage through AHRI’s new ClinicLink system.
- Capturing of clinic attendance in the PIP area. AHRI implemented the ClinicLink system in 2017, to collect the date and reason for attendance of all individuals who attend one of the 11 clinics in the PIP area. Consenting individuals who are referred to care after HCT, or other screening tests, and do not attend a clinic within 10 days are sent a reminder text message; those who have still not attended within 30 days are contacted by telephone by a trained counsellor, and encouraged to attend for care.
- Linkage to routine data sources. An important feature of PIP is the ability to link the surveillance data with a range of routine data sources (Table 2; Supplementary Figure S2, available as Supplementary data at *IJE* online). The linkage algorithm is initially deterministic, with successive steps based on five key linkage variables (South African national identification number, first name, surname, sex and date of birth), followed by probabilistic matching on the same five variables with manual verification. Current linked data sources include the national HIV care database (TIER.net) with records for all individuals on ART at 17 clinics in the sub-district. In 2018, there were 64 785 individuals with a record in TIER.net, of whom 17 531 (27%) were members of PIP households. Among 18 662 individuals in the PIP cohort who ever tested positive in the HIV surveillance, 8411 (45%) had a record in TIER.net. Other linked data sources include the Hospital Information System, containing data on all admissions to Hlabisa hospital (the local district hospital) since 2010. Permission from the relevant government authorities has been granted for access to data from the National Health Laboratory Service (NHLS), electronic TB registers (now part of TIER.net), and records from the Departments of Health, Social Welfare, Home Affairs and Education. Linkage to these data sources is anticipated in the future.

**Table 1:** Data collected at household and individual surveys

Area	Types of information	Frequency	Eligibility criteria
**Household**			
Household demographics	Household members (dates of birth, sex, relationship to household head, marital status, residency status), births, deaths, in- and out-migration	Three times annually since 2000	All households in surveillance area
Household socio-economic data	Household assets, household infrastructure (water, sanitation, electricity), food security, experience of violence, household expenditure	2001, 2003, 2005, annual thereafter, except 2008 2017	All households in surveillance area
Individual socio-economic data	Education, employment	Annual since 2003	All individuals who are members of households in the surveillance area
Government grants	Receipt of government grants for old age, disability, child support, etc.	2003, 2005, 2006, annual thereafter	All individuals who are members of households in the surveillance area
**Individual**			
HIV status	HIV status (from anonymized testing) Self-reported: Knows HIV status When last tested When last tested negative/positive Currently on ART	Annual since 2003 Annual since 2006 Annual since 2010 Annual since 2017	2003–2006: women 15–49 years and men 15–54 years resident in surveillance area After 2006, all residents aged ≥15 years
Sexual behaviour	Pregnancy history (women) Number of children fathered (men) Contraceptive use (women) Sexual activity Attitudes to condom use	Annual since 2003	2003–2008: women 15–49 years and men 15–54 years resident in surveillance area After 2008, all residents aged ≥15 years
General health	Self-reported: Hospitalized in past year Hypertension diagnosis/treatment Diabetes diagnosis/treatment TB diagnosis/treatment Circumcized (men)	Annual since 2009	All individuals aged ≥15 years resident in surveillance area
Biomeasures	Height/weight Blood pressure	2003, 2010	All individuals aged ≥15 years resident in surveillance area

**Table 2:** Description of data available in linked data sources

Data source	Types of information	Description
TIER.net	Clinic visits for ART care; viral load; CD4 counts, ART regimen at initiation; changes in ART regimen	Electronic patient records for individuals on ART in any of 17 clinics in the Hlabisa health sub-district and Hlabisa hospital since 2004
Hospital information system	Admission date; discharge date; ward admitted to; ICD10 diagnosis; discharge status	All admissions to Hlabisa hospital since 2010, except for routine deliveries
ClinicLink	Date of visit; reason for visit	Individuals attending one of 11 clinics serving the PIP surveillance area since 2017
Vukuzazi clinical phenotype cohort	Anthropometric data; blood pressure; HbA1c; chest X-ray; sputum culture/Xpert; HIV viral load (if HIV positive)	All resident household members aged ≥15 years in the PIP surveillance area (2018/2019)

HbA1c refers to glycated haemoglobin.

**Figure 3:**
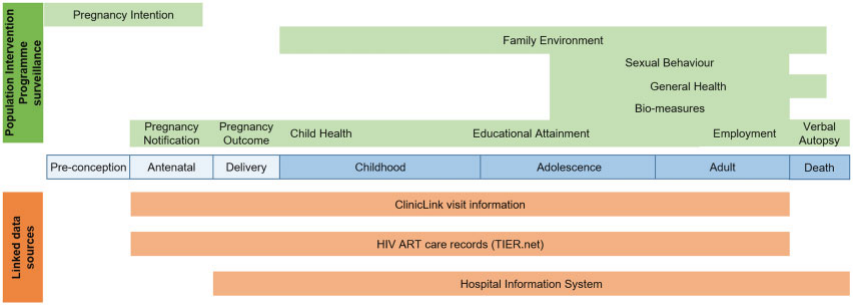
Data gathered in PIP surveillance (green) and linked data sources, demonstrating the potential to address a range of health research questions across all the stages of the life course

In 2018/19, AHRI established the Vukuzazi Clinical Phenotype Cohort nested within PIP, which included multi-disease screening and collection of a range of bio-measures. The linkage of multiple data sources with information collected in the annual PIP surveillance or ancillary studies enables a ‘health across the lifespan’ approach to research (Figure 3).

Ethical approval for the PIP surveillance, ClinicLink, and linkage of routine data sources was granted by the Biomedical Research Ethics Committee, University of KwaZulu-Natal, South Africa (BE290/16). Separate written informed consent is obtained for the household survey, the individual surveys and HCT. Informed consent is also obtained for facilitated linkage to care. An R50 (∼US$3) unconditional food gift voucher is given to PIP households during the annual home visit; no other gifts or incentives are given for participation. Clinic attendees provide informed consent to record their visit in ClinicLink. A waiver of individual consent for data linkage to the routine data sources has been obtained. The linked datasets are anonymized such that individuals’ confidentiality will not be compromised through the linkage. Participants provide informed consent to share their anonymized data with researchers.

## What has it found? Key findings and publications

Over 800 papers, covering a range of health outcomes, have been published since 2008 using data collected from the PIP cohort. Much of this work has been on the epidemiology of HIV, including prevention and treatment. Analyses of PIP data have shown the tremendous effect of ART on life expectancy: adult life expectancy in 2003 (the year before ART became available in the public-sector clinics) was 49.2 years; by 2011, it had increased to 60.5 years.^5^ PIP data have also provided estimates of the effects of community- and household-level ART coverage, and of population HIV viral load, on the risk of HIV acquisition.^11–13^ Increased community-level ART coverage was also shown to be associated with a decreased risk of TB disease.^14^

Papers have been published on temporal trends in detectable HIV viraemia,^15^ the HIV care cascade^16^ and the effect of ART scale-up.^17^ Studies have examined behavioural risk factors for HIV incidence, including the effect of age-disparate relationships,^18^^,^^19^ male circumcision,^20^ migration patterns^21–23^ and changes in sexual behaviour.^24^

AHRI’s increased focus on interventions included a CRT to increase HIV testing and treatment uptake among men,^25^ a CRT to evaluate peer-delivery of HIV self-testing,^26^ a stepped-wedge CRT to improve antenatal care,^27^ and evaluation of a large combination HIV prevention programme among young women.^28^^,^^29^

Recent studies have also examined health service access, linkage to care and clinical outcomes.^30^^,^^31^ A study of the impact of COVID-19 lockdown showed no decrease in visits to primary health care clinics.^32^ PIP is also a member of the Analysing Longitudinal Population-based HIV data on Africa (ALPHA) network and has contributed to many multi-site analyses across sub-Saharan Africa.^33^

## What are the main strengths and weaknesses?

A major strength of the PIP cohort is its location and size: it is situated at the centre of dual HIV and TB epidemics and is one of the world’s largest population-based HIV surveillance studies (with >3500 prospectively documented HIV seroconversions), with wide-ranging longitudinal information measured over 20 years. The household survey has had a consistently high response rate, providing nearly complete data on births, deaths and migration patterns. Data collection is carefully monitored to maintain quality, and detailed documentation is available for all datasets.

Another key strength is the ability to link the cohort data to a wealth of routine data sources, providing objective information on HIV treatment, morbidity, health service usage and a range of health outcomes. As such, the PIP cohort provides a powerful resource for HIV, TB and NCD research, and a strong platform to accurately measure population dynamics, disease burden, and use of health and other services, and to implement and evaluate individual- and population-level interventions.

An important limitation is the comparatively low response rate on the individual components of the annual survey. However, the existence of a comprehensive sampling frame, based on the household survey, makes it possible to examine the extent to which representativeness is maintained in the individual components, and to quantify the effect of potential biases from non-participation.^34^ Furthermore, most survey data are self-reported and record linkage with routine data is imperfect, so some data may fail to be (or be incorrectly) linked.

## Can I get hold of the data? Where can I find out more?

PIP data can be accessed through the AHRI data repository (https://data.ahri.org/index.php/home), after self-registration and completion of a short data-use agreement form (available online). Data documentation, including questionnaires, technical documents and data dictionaries, is available in the repository.

## Supplementary data

Supplementary data are available at *IJE* online.

## Funding

This work was supported by the Wellcome Trust through core funding to the Africa Health Research Institute (082384/Z/07/Z), and by the South African Department of Science and Innovation through the South African Population Research Infrastructure Network (SAPRIN) hosted by the South African Medical Research Council and Wellcome.

## Supplementary Material

dyaa264_Supplementary_Data
