# Supplementary material for: Cohort Profile Update: Africa Centre Demographic Information System (ACDIS) and population-based HIV survey
Source: Int J Epidemiol. 2021 Jan 12;50(1):33–4. doi: 10.1093/ije/dyaa264 (PMC7938501; doi:10.1093/ije/dyaa264)

**Supplementary Figure S1. HIV prevalence by age and sex among residents participating in 2018 serosurvey**

**
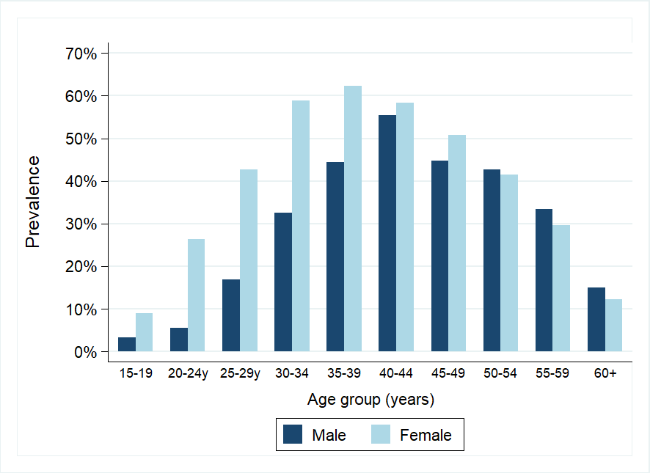
**

**Supplementary Figure S2. Data gathered in Population Intervention Platform (PIP) demographic and HIV surveillance and linked data sources, with years of collection and total number of individuals in each database since inception, and the number who have been linked to household members in the PIP surveillance area.**


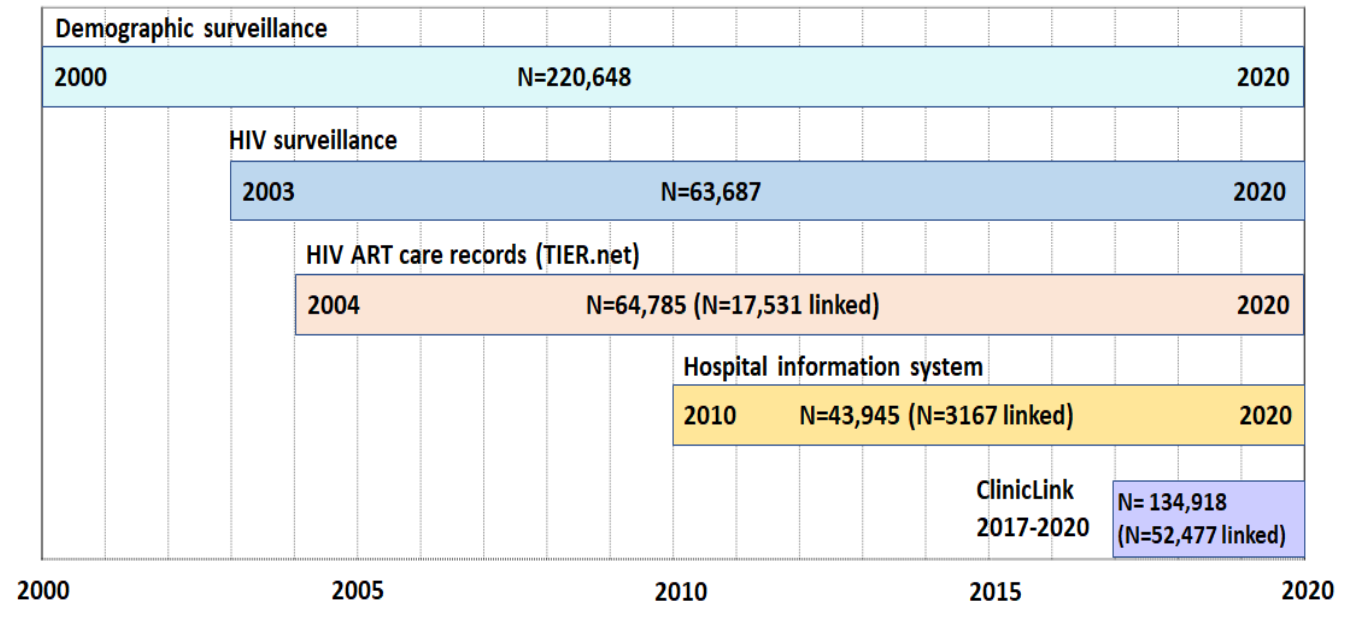

Supplement: dyaa264_Supplementary_Data [file dyaa264_supplementary_data.zip › ije-2020-07-1336-File007.docx]
