# Supplementary material for: Cohort Profile Update: Africa Centre Demographic Information System (ACDIS) and population-based HIV survey
Source: Int J Epidemiol. 2021 Jan 12;50(1):33–4. doi: 10.1093/ije/dyaa264 (PMC7938501; doi:10.1093/ije/dyaa264)
Supplement: dyaa264_Supplementary_Data [file dyaa264_supplementary_data.zip › Supplementary tables_submitted_17Jul2020.docx]

**Supplementary Table S1. Mid-year populations of PIP cohort from 2000 to 2018, and proportion of members who are resident and non-resident**

| **Year** | **Non-resident ( row %)** | **Resident ( row %)** | **Total** |
| --- | --- | --- | --- |
| 2000 | 25,365 (28.3%) | 64,109 (71.7%) | 89,474 |
| 2001 | 21,568 (24.2%) | 67,623 (75.8%) | 89,191 |
| 2002 | 21,520 (24.2%) | 67,563 (75.8%) | 89,083 |
| 2003 | 22,022 (25.0%) | 65,913 (75.0%) | 87,935 |
| 2004 | 23,299 (26.4%) | 64,920 (73.6%) | 88,219 |
| 2005 | 24,163 (27.1%) | 64,939 (72.9%) | 89,102 |
| 2006 | 25,132 (27.8%) | 65,384 (72.2%) | 90,516 |
| 2007 | 25,487 (27.8%) | 66,134 (72.2%) | 91,621 |
| 2008 | 25,496 (27.7%) | 66,551 (72.3%) | 92,047 |
| 2009 | 25,401 (27.2%) | 67,877 (72.8%) | 93,278 |
| 2010 | 25,771 (27.2%) | 68,924 (72.8%) | 94,695 |
| 2011 | 26,456 (27.7%) | 69,208 (72.3%) | 95,664 |
| 2012 | 27,740 (29.0%) | 67,851 (71.0%) | 95,591 |
| 2013 | 28,651 (30.1%) | 66,579 (69.9%) | 95,230 |
| 2014 | 29,194 (30.6%) | 66,347 (69.4%) | 95,541 |
| 2015 | 29,892 (31.1%) | 66,182 (68.9%) | 96,074 |
| 2016 | 30,410 (32.1%) | 64,370 (67.9%) | 94,780 |
| 2017 | 37,505 (25.9%) | 107,554 (74.1%) | 145,059 |
| 2018 | 39,348 (27.7%) | 102,731 (72.3%) | 142,079 |

**Supplementary Table S2. Participation rates in each component of the annual individual survey, 2013-2018**

| **Year** | **Eligible^1^** | **Contacted (% of eligible)** | **N consenting to each component (% of contacted)** | | | |
| --- | --- | --- | --- | --- | --- | --- |
|  |  |  | **HIV serosurvey** | **General health** | **Sexual behaviour** | **HIV rapid test** |
| 2018 | 54,449 | 39,684 (72.9%) | 19,542 (49.2%) | 20,213 (50.9%) | 11,363 (28.6%) | 11,691 (29.5%) |
| 2017 | 53,400 | 42,958 (80.4%) | 18,015 (41.9%) | 13,731 (32.0%) | 6070 (14.1%) | 10,874 (25.3%) |
| 2016 | 33,975 | 28,816 (84.8%) | 14,726 (51.1%) | 15,344 (53.2%) | 8632 (30.0%) | N/A^2^ |
| 2015 | 32,201 | 27,789 (86.3%) | 13,120 (47.2%) | 15,218 (54.8%) | 7609 (27.4%) | N/A |
| 2014 | 33,009 | 25,397 (76.9%) | 9565 (37.7%) | 13,017 (51.3%) | 7287 (28.7%) | N/A |
| 2013 | 32,956 | 25,577 (77.6%) | 9922 (38.8%) | 14,948 (58.4%) | 8272 (32.3%) | N/A |

^1^Resident member of a household in the surveillance area and aged ≥15 years. ^2^Annual home-based HCT offered from 2016-onwards.
