# Supplementary figures and images for: Cohort Profile Update: Africa Centre Demographic Information System (ACDIS) and population-based HIV survey
Source: Int J Epidemiol. 2021 Jan 12;50(1):33–4. doi: 10.1093/ije/dyaa264 (PMC7938501; doi:10.1093/ije/dyaa264)

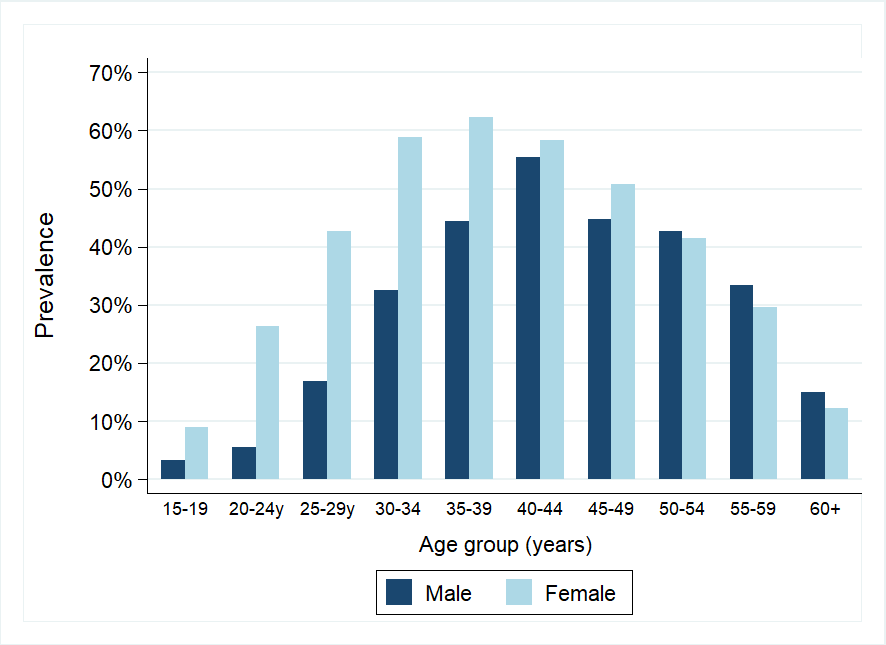

Supplement: dyaa264_Supplementary_Data [file dyaa264_supplementary_data.zip › ije-2020-07-1336-File008.tif]

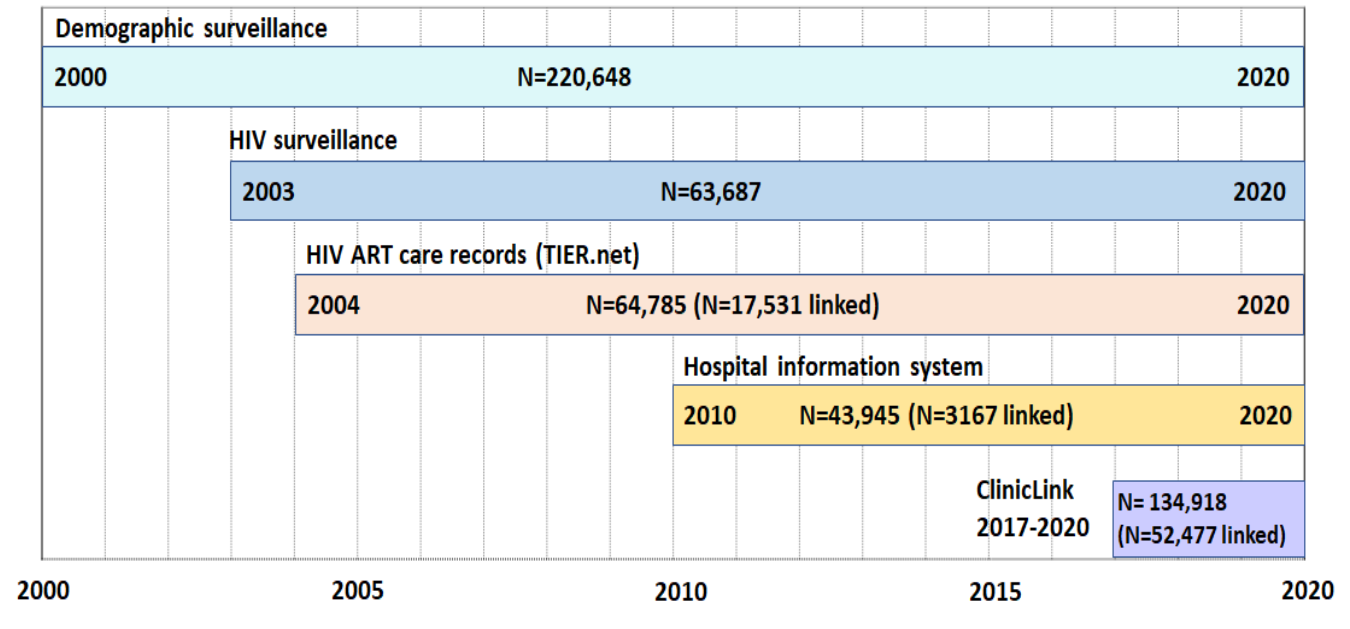

Supplement: dyaa264_Supplementary_Data [file dyaa264_supplementary_data.zip › ije-2020-07-1336-File009.tif]
